# Use of Multicenter Data in a Large Cancer Registry for Evaluation of Outcome and Implementation of Novel Concepts

**DOI:** 10.3389/fonc.2017.00234

**Published:** 2017-09-29

**Authors:** Gabriele Schubert-Fritschle, Stephanie E. Combs, Thomas Kirchner, Volkmar Nüssler, Jutta Engel

**Affiliations:** ^1^Munich Cancer Registry (MCR) of the Munich Tumour Centre (TZM), Institute for Medical Information Processing, Biometry and Epidemiology (IBE), University Hospital of Munich, Ludwig-Maximilians-University (LMU), Munich, Germany; ^2^Munich Tumour Centre (TZM), Medical Faculties, Ludwig-Maximilians-University (LMU) and the Technical University of Munich (TUM), Munich, Germany; ^3^Department of Radiation Oncology, Technische Universität Munich (TUM), Klinikum rechts der Isar, Munich, Germany; ^4^Department of Radiation Sciences (DRS), Institute for Innovative Radiotherapy (iRT), Helmholtz Zentrum Munich, Oberschleißheim, Germany; ^5^Deutsches Konsortium für Translationale Krebsforschung (DKTK), Partner Site Munich, Munich, Germany; ^6^Institute for Pathology, Ludwig-Maximilians-University (LMU), Munich, Germany

**Keywords:** cancer incidence, cancer mortality, survival, trends, data analysis, quality assurance, comparative effectiveness research

## Abstract

Large clinical cancer registries (CCRs) in Germany shall be strengthened by the German Social Code Book V (SGB V) and implemented until the end of 2017. There are currently several large cancer registries that support clinical data for outcome analysis and knowledge acquisition. The various examples of the Munich Cancer Registry outlined in this paper present many-sided possibilities using and analyzing registry data. The main objective of population-based cancer registration within a defined area and the performance of outcomes research is to provide feedback regarding the results to the broad public, the reporting doctors, and the scientific community. These tasks determine principles of operation and data usage by CCRs. Each clinical department delivers its own findings and applied therapy. The compilation of these data in CCRs provides information on patient progress through the regional network of medical care and delivers meaningful information on the course of oncological diseases. Successful implementation of CCRs allows for presenting the statistical outcomes of health-care delivery, improving the quality of care within the region, accelerating the process of implementing innovative therapies, and generating new hypotheses as a stimulus for research activities.

## Regional CCRs—Instruments for Clinical and Epidemiological Research

According to the 1995 German law regarding the regulating of cancer registration (Cancer registry law, Krebsregistergesetz—KRG 1995), German states were required to establish cancer registries until January 1999. All German states complied with this regulation and generated comprehensive epidemiological cancer registrations. Over time, there has been increasing precision in the estimation of cancer incidence and mortality by the German Society for Epidemiological Cancer Registries in Germany (GEKID) (1) and the Centre for Cancer Registry Data (ZFKD) at the Robert Koch-Institute (RKI) (2). In addition, data from nine German regions are currently published in the WHO publication Cancer Incidence in Five Continents, Vol. X (3), and 23% of EUROCARE-5 (4) data originate from Germany.

Population-based cancer registries are important instruments for epidemiological reference. Epidemiology involves the analysis of health and illness or, more generally, the dynamics, causes, and consequences of the health status of a defined population (5). Cancer will affect more than 40% of all people globally. In Germany alone, approximately 477,000 persons per year are diagnosed with cancer, and 221,000 persons die each year from cancer (6).

The few indices, however, that may be estimated by epidemiological cancer registries are not sufficient to describe the complex structures, care, and outcomes of cancer diseases. Therefore, it is necessary to obtain evidence on cancer subtypes, various outcomes of cancer care, clinical experiences, and knowledge origination, all of which require population-based clinical data collected by way of CCRs and then analyzed and published by the registries in cooperation with their clinical partners.

Therefore a national law, the Krebsfrüherkennungs- und -registergesetz (KFRG), which generated basic conditions for population-based CCRs in all regions of Germany (SGB V §65c), was enacted in April 2013. The KFRG requires that cancer data be recorded in CCRs countrywide, but in small regions (federal state or parts of it), in accordance with standardized definitions [see common German oncological basic dataset and its supplementary organ-specific modules edited by ADT (Arbeitsgemeinschaft Deutscher Tumorzentren, working group of German cancer centers) and GEKID]. Data acquisition must be completed relative to cases with defined ICD-10 codes and items of ADT-oncological data sets within a defined region. Each federal state has to legislate specific details (e.g., data protection) by its own.

Clinical cancer registries may provide useful data on cancer diseases and cancer care if all doctors and hospitals within a defined region and within defined fields, such as surgery, pathology, radiotherapy, and systemic oncology, prepare and submit all independent and cross-sectoral findings and therapies from the primary diagnosis through the course of the disease. Data are checked, coded, and compiled in CCRs, and data management is completed using follow-up information, including date and cause of death. Based on this structure, feedback may be realized in the manner postulated by law.

## The Munich Cancer Registry (MCR)—Organization and Structure of a Multicenter Data Pool

**Figure 1:**
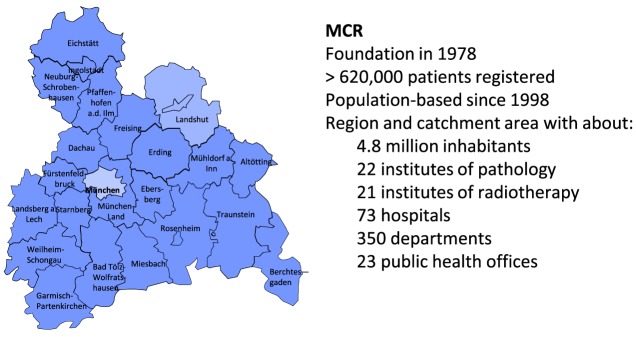
The Munich Cancer Registry (MCR)—catchment area and key data (7).

The MCR is the population-based clinical cancer registry of Upper Bavaria and one region of Lower Bavaria (Southern Germany) (7). Since 1978, the registry’s catchment area has been enlarged twice. In 2002, it was increased to 2.3 million inhabitants, and in 2007, it was increased to 4.5 million inhabitants. It currently includes more than 4.8 million inhabitants (Figure 1).

Pathology reports of solid tumors from all pathology laboratories in this catchment area are available. From these reports, the total number of cancer patients in the region is systematically assessed and the main prognostic factors were ascertained. In parallel, clinicians complete standardized forms concerning patients’ domicile, age, primary disease characteristics such as TNM-stage, histology, grade, as well as therapies or deliver these data online to the MCR.

**Figure 2:**
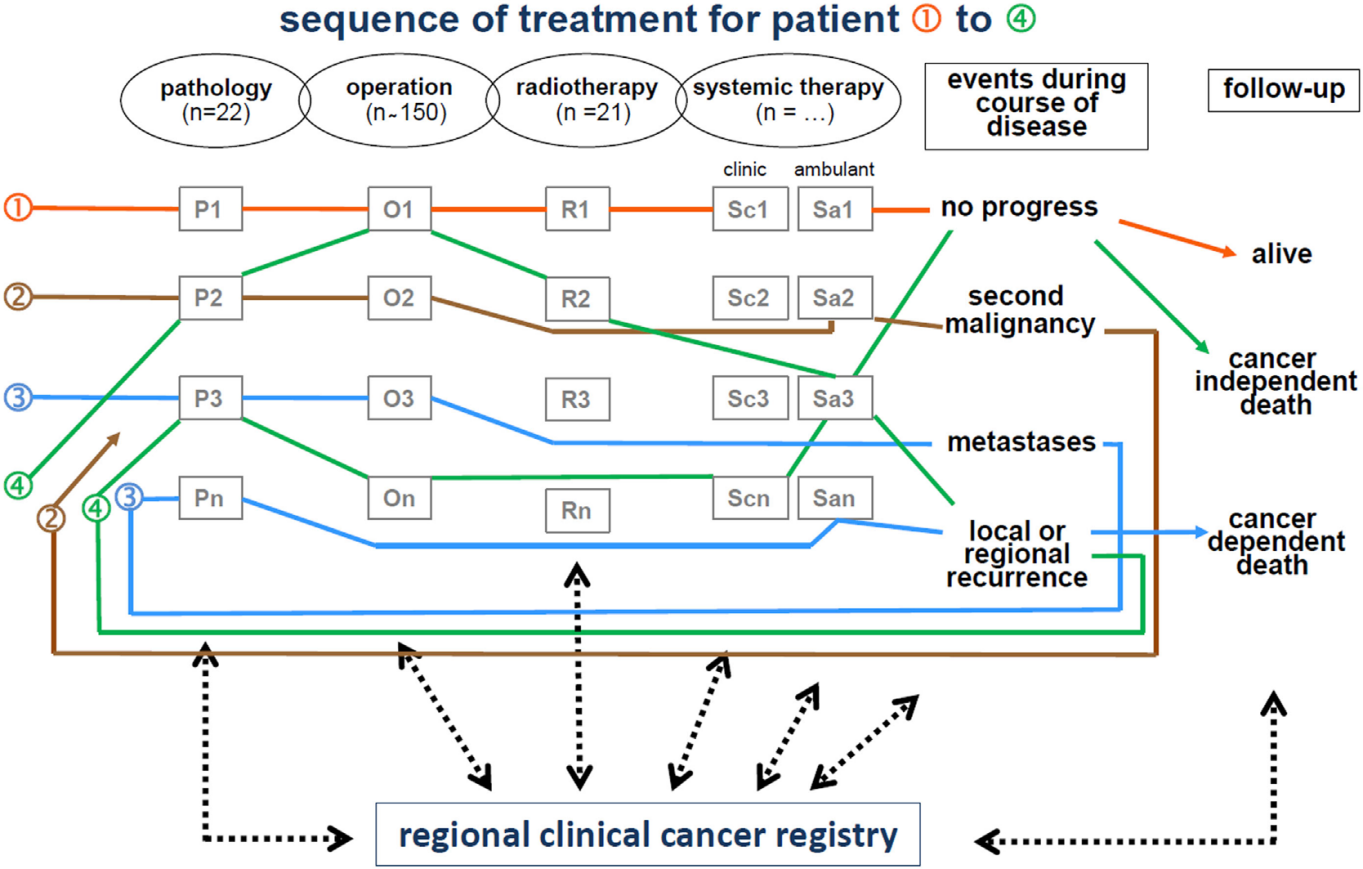
Cancer registration by interdisciplinary and cross-sectoral procedure.

The life status of patients diagnosed with cancer is maintained by clinicians and is systematically updated by the MCR through death certificates. Figure 2 displays the interdisciplinary and cross-sectoral documentation of course of cancer disease.

All data and clinical findings during the course of the disease (e.g., local or regional recurrence, metastases, and death) are coded according to the guidelines of the International Agency for Research on Cancer. Tumors are classified in compliance with the staging criteria of the TNM Classification of Malignant Tumors (8). There are approximately 350 departments in approximately 70 hospitals in the cooperating network of the MCR. Currently, more than 25,000 new cases are registered each year. Within the smaller catchment area, there were approximately 11,000 new cases reported annually in 1998, which increased to approximately 25,000 annually by 2015. Accordingly, there were more than 360,000 cancer cases registered in the MCR from 1998 to 2015 (Table 1).

**Table 1:** Malignancies by year of diagnosis from 1998 to 2015 defined by KFRG.

ICD10-diagnosis	C00-C97 without C44 and C77-C79	D00-D09 without D04	D32-D33 D35.2-4	D39.1 D41.4 D42-D43 D44.3-5 D45-D46 D47.1/3-5	Total	Portion death certificate-only (DCO)		Portion children	
			
Year	*n*	*n*	*n*	*n*	*n*	*n*	%	*n*	%
1998	10,682	564	37	79	11,362	1,355	10.6	69	0.6
1999	10,744	611	44	90	11,489	1,281	10.0	64	0.6
2000	10,619	638	94	101	11,452	1,446	11.2	62	0.5
2001	11,075	650	204	100	12,029	1,458	10.8	57	0.5
2002	18,336	1,009	297	176	19,818	3,253	14.0	94	0.5
2003	18,371	1,143	292	167	19,973	2,729	12.0	109	0.5
2004	18,751	1,489	268	181	20,689	2,556	10.9	121	0.6
2005	19,066	1,592	298	211	21,167	2,287	9.7	146	0.7
2006	19,485	1,630	307	237	21,659	1,995	8.4	116	0.5
2007	22,407	1,928	398	322	25,055	2,406	8.7	154	0.6
2008	22,943	2,086	376	329	25,734	2,233	7.9	156	0.6
2009	22,787	2,170	383	363	25,703	2,085	7.5	118	0.5
2010	22,520	2,376	256	348	25,500	2,158	7.8	145	0.6
2011	22,743	2,561	397	359	26,060	2,069	7.3	149	0.6
2012	22,734	2,480	374	294	25,882	2,008	7.2	168	0.6
2013*	21,737	2,660	207	294	24,898	1,991	7.4	136	0.5
2014*	17,681	2,126	174	203	20,184	2,003	9.0	63	0.3
2015*	14,218	1,656	29	120	16,023	1,610	9.1	20	0.1
Total	326,899	29,369	4,435	3,974	364,677	36,923	9.2	1947	0.5

*Without DCO-cases (portion from sum of columns total, DCO and children)*.

*Without children <18 years (portion from sum of columns total + children)*.

*Without non-melanotic skin cancer (C44, D04), secondary malignancies (C77-C79)*.

All patients are followed actively and prospectively to make the database as complete as possible. Over time, approximately 50% of the patients have deceased. The course of the disease in the other 50% is continuously being adjusted by including information regarding disease progression and life status. Furthermore, the percentage of death certificate-only cases decreased from 10 to 7% from 1998 to 2015.

The diagnoses of cancers with high incidence rates, such as breast or colorectal cancer, range from 3,500 to 4,000 per year. While the catchment area is large, only a small number of patients with rare cancers, such as vulvar cancer or Hodgkin’s lymphoma, is expected. This quantity structure must be considered in the data analysis and study design. Nonetheless, even given the cooperation of several institutions and the aggregation of time periods, there remain an insufficient number of patients with rare cancers to conclude reliable survival analyses or to conduct multivariate statistical methods.

## Feedback Regarding Results

**Figure 3:**
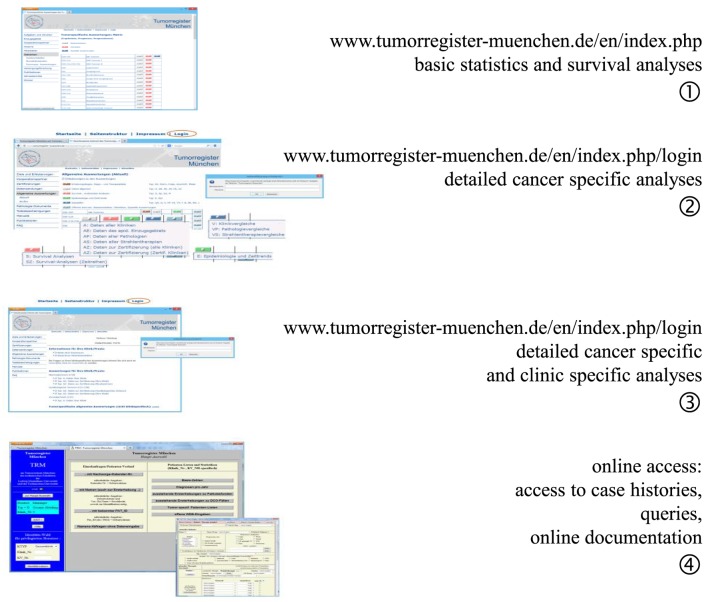
Four levels of feedback *via* the Internet.

One of the elementary tasks of CCRs is to provide information regarding patients’ cancers to the cooperating partners, doctors, hospitals, public, and most especially, to the patients and their relatives. The MCR developed four levels of data presentation, all of which can be accessed *via* the Internet (Figure 3).

Level 1 provides open access information on age distribution, incidence, mortality, and survival as ordered by ICD-10 (C- and D-diagnoses) for all interested persons (9). Access to level 2 information is restricted by password to cooperators and authorized persons. Level 2 provides special analyses of the whole catchment area and is limited to the main ICD-10 cancer diagnoses. The statistics and results presented in level 3 are restricted to the cohort of patients of a single hospital and may be accessed using a unique hospital password. Level 1 to level 3 presents aggregated statistics with a varying degree of detail. Finally, doctors with special personal identification are allowed online access to the MCR database in level 4. Basic queries may be performed on single patients or patient groups with defined characteristics, and it is possible to perform online documentation of cancer patients.

## Use of Multicenter Data to Evaluate Outcomes and Implement Novel Concepts

**Figure 4:**
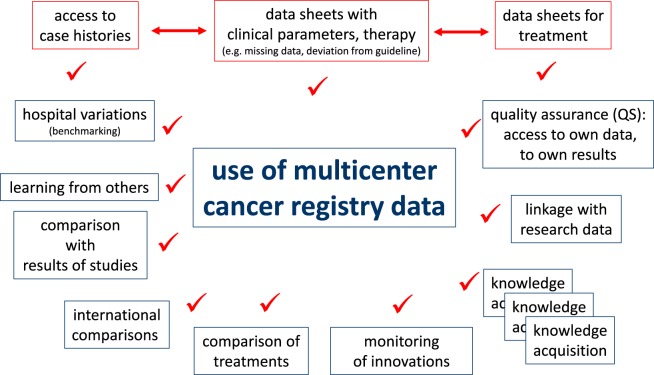
Aspects of data use and research objectives.

Cancer registry data may be used in various clinical and scientific applications. Clinicians must have access to case histories for daily patient care or for evaluation of the course of disease for single patients. A precondition for clinical QA measures is to ensure data correction and completion of documents that gather information such as disease parameters and types of therapies. Certain questions are intended for patient cohorts with comparable diagnostics or treatments. The demands on an individual level are described in the upper portion of Figure 4.

Descriptive and analytical statistics are essential for certain scientific applications. For example, the analyses of hospital variations are useful for benchmarking, providing information feedback required for QA and reproducing published results using epidemiological data from the CCR for CER. These and other examples are outlined in the lower portion of Figure 4. The duties and responsibilities of CCRs can be defined through comparisons of hospitals, analyses of certification audits, comparisons to cancer-specific guidelines, assessments of regional and time trends, and individual benchmark results of clinical study outcomes.

Caution should be taken when interpreting the results, as data from single cooperating hospitals have various types of bias and lack representation. Thus, for proper comparisons, CCRs should provide statistics that include averages of epidemiological results from general clinical data and data on the course of the disease that are aggregated by different characteristics. In this way, comparisons can be made and single hospital results can be appropriately interpreted.

## Outcome Evaluation

Additional clinical data are required for QA and CER in oncology to increase the explanatory power attained by representing a defined population. With relevant positive and negative deviations found through multivariate data analyses, the following examples illustrate the various uses of CCR data to evaluate outcomes.

### Estimation of Prognosis

A cancer prognosis is important not only for evaluating outcomes but also for the patients’ information. The main criterion of prognosis is survival, which is calculated as overall survival (OS), which includes all deceased individuals, and relative survival (RS). RS is the ratio of the observed survival rate to the expected survival rate. RS may be interpreted as cancer survival after correcting for other causes of death; therefore, it is used to estimate cancer-specific survival. The expected survival time of age-matched individuals is calculated according to the Ederer II method using life tables of the German population (10).

**Figure 5:**
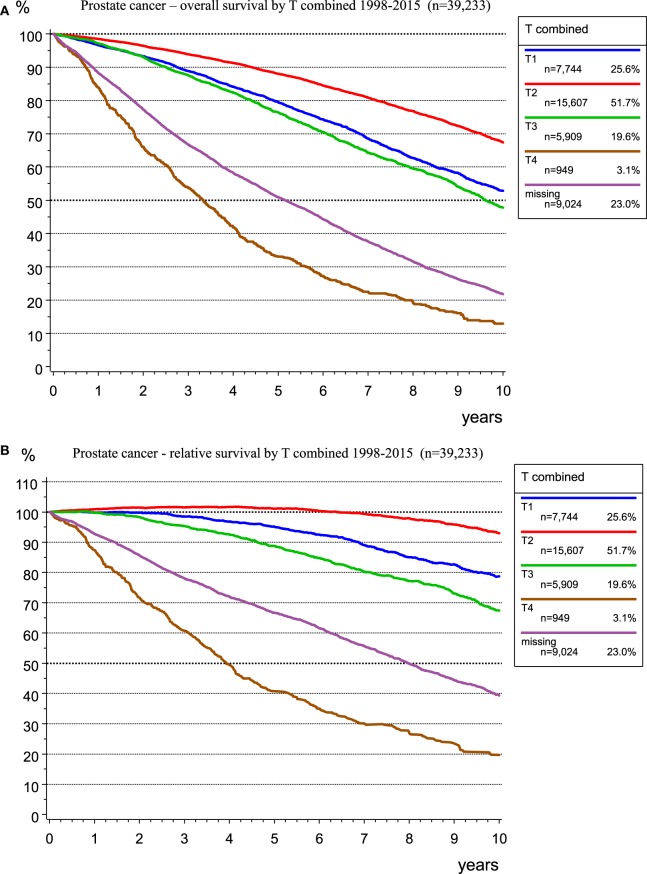
Observed overall **(A)** and relative **(B)** survival for prostate cancer by T-categories for 39,233 patients (1998–2015).

There is a good prognosis for people with prostate cancer, a disease in older men with a median age of 69 years, especially in the T1 and T2 categories. Figure 5 shows OS (Figure 5A) and RS (Figure 5B) as estimator of cancer-specific survival. The survival rate (>100%) for patients with T2 tumors during the first 7 years after diagnosis is better than the mean survival rate of the German male population. The relative 5-year survival rates are 101.1 and 95.1% for the T2 and T1 categories, respectively, primarily due to incidental carcinomas. Figure 5 shows the effect of calculating RS, which accounts for the mean life expectancy of the German male population.

**Figure 6:**
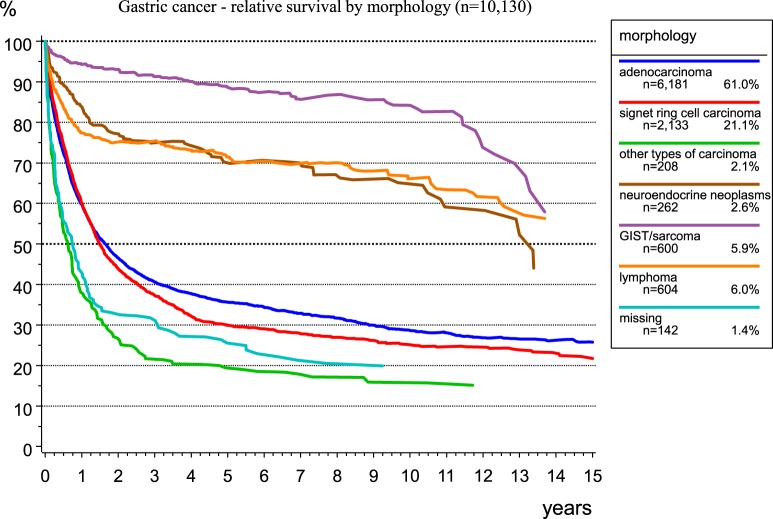
Relative survival of patients with gastric carcinoma by morphology.

The morphological verification of tumors delivers important information for treatment planning and prognosis estimation. Figure 6 presents the spectrum of morphology and the frequency of morphological types of gastric cancer. As RS largely depends on morphology, there are better results for GIST and neuroendocrine neoplasms. The relative 5-year survival rates for patients with stomach adenocarcinoma, signet ring cell carcinoma, and GIST/sarcoma are 35.6, 29.9, and 88.6%, respectively.

### Benchmarking

There may be selection bias within single hospitals that influences outcomes. Therefore, the results of single institutions must be compared to each other using measures based on summary population-based data. Accordingly, these results may be interpreted as the mean epidemiological values.

**Figure 7:**
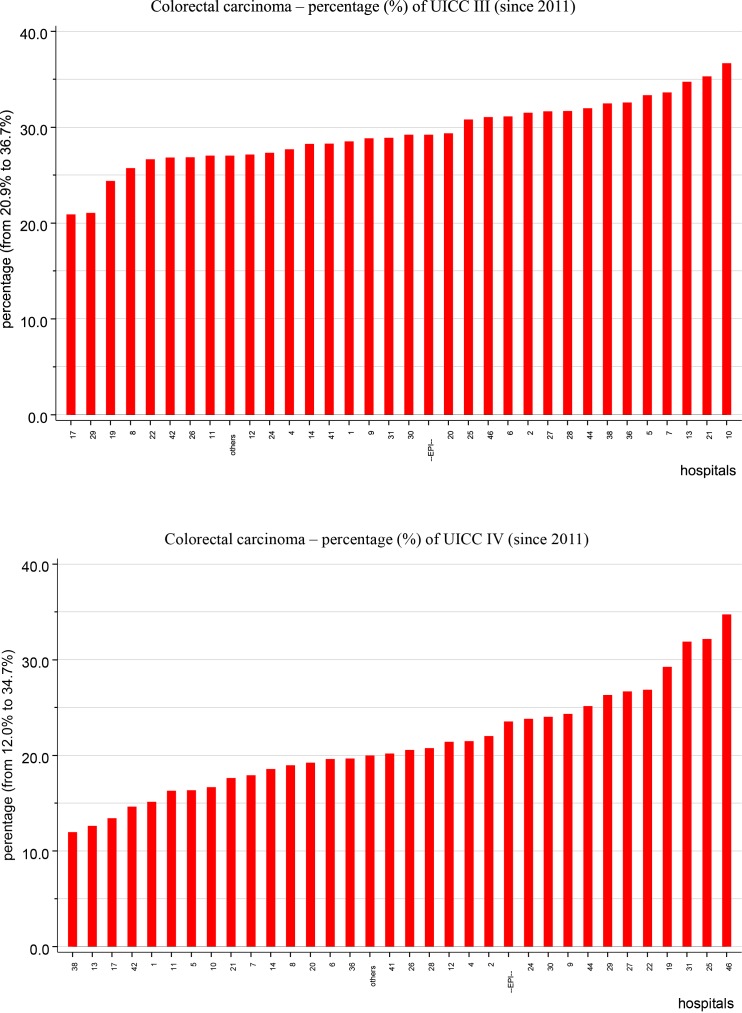
Various percentages of colorectal cancer UICC III and IV for co-operating clinics.

Figure 7 presents two diagrams of the percentages of UICC stage III and IV colorectal cancer, where one red bar indicates one co-operating hospital. For UICC stage III colorectal cancer, the upper diagram reveals a variation between 20.9 and 36.7%, with an epidemiological mean of 29.2%. For UICC stage IV colorectal cancer, the lower diagram shows a variation between 12.0 and 34.7%, with an epidemiological mean of 23.5%.

This example of clinic-specific variation emphasizes the use of multivariate statistical methods, such as proportional hazard models, to adjust not only for multiple prognostic parameters but also for clinical variations.

## Implementation of Novel Concepts

The implementation of novel concepts in cancer care is a continuous process that relies on the presumption of results from research and randomized clinical studies as well as reliable evidence from observational data (e.g., provided by population-based CCRs). Oncological guidelines compile and periodically actualize the state of the art of diagnostics and treatment. CER proves the degree of implementation and the effectiveness of the applied measures.

### Breast Cancer

**Figure 8:**
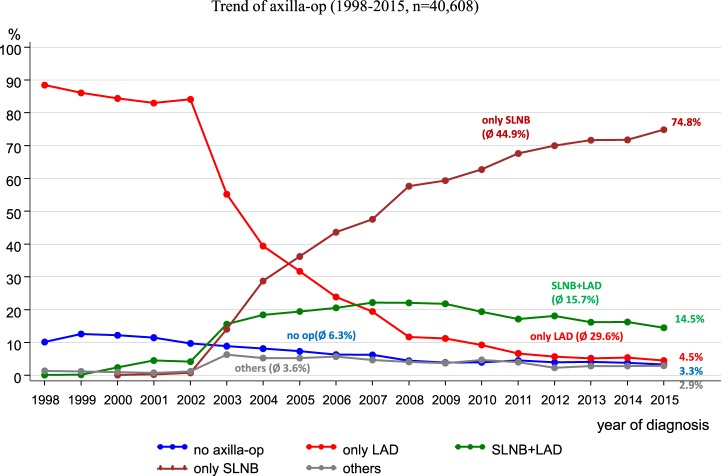
Trends of various types of axilla operations.

Since 2008, the German S3 guideline for breast cancer has recommended a sentinel lymph node biopsy (SLNB) (11). Figure 8 reveals the implementation of the SLNB parallel to the trends of other axilla operations within the catchment area of the MCR. When the guideline was published in 2008, SLNBs were being practiced in more than 50% of all axilla operations. In 2015, SLNBs were performed in 74.8% of all axilla operations.

From 1998 to 2015, lymphadenectomies during the observation period decreased from 88.5 to 4.5%, respectively.

**Figure 9:**
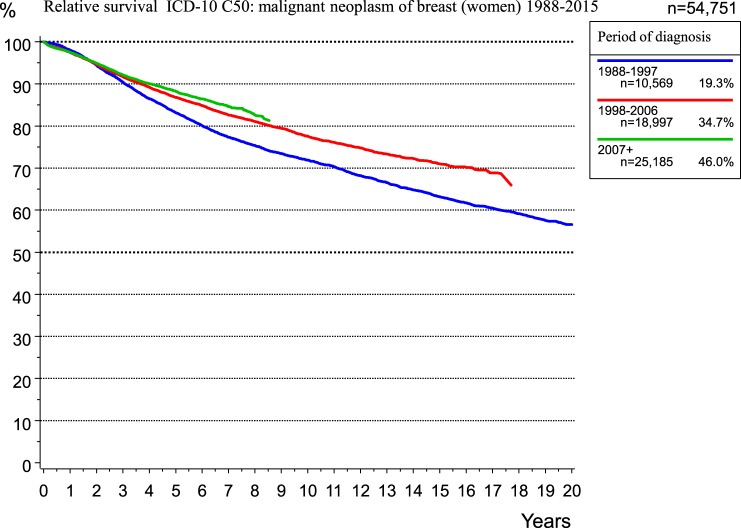
Relative survival for breast cancer.

Additionally, during this time, adjuvant and neoadjuvant therapy was refined and intensified. Accordingly, the RS rate for breast cancer patients within the MCR region increased from 1998 to 2015 (Figure 9).

### Vulva Carcinoma

An article published about a less invasive local lymph node surgery for squamous cell vulvar carcinoma (12) reported that in an analysis of 1,133 patients diagnosed between 1998 and 2013, there were significant decreases in complete vulvectomies and inguinal lymph node surgeries. Moreover, the change in therapy to less radical procedures did not negatively affect the time to local and lymph node recurrence, OS, or RS.

This publication is an example of the limits of evidence-based medicine, but it also indicates that population-based CCRs with a large catchment area of about five million inhabitants and a multicenter cooperation structure such as the MCR still deliver for small cohorts of rare cancers such as vulvar carcinoma. An important advantage of larger CCRs is its possibility to deal with rare cancers.

### Lung Cancer

**Figure 10:**
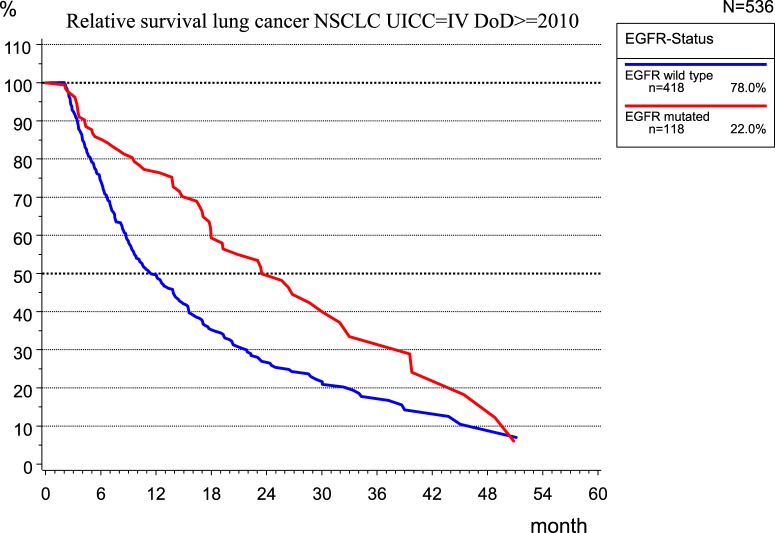
Relative survival for non-small cell lung cancer, date of diagnoses ≥2010, UICC IV.

The implementation of new therapeutic concepts in patient care requires the evaluation of their effects on outcomes. For therapy planning and predicting the prognosis of UICC stage IV non-small cell lung cancer (NSCLC), the role of the EGFR mutation (EGFR mutated) was examined in 536 patients of the MCR (Figure 10) because the mutation of EGFR is a good predictor of the effectiveness of tyrosine kinase inhibitors (13).

While there is no validation of the type of therapy applied, the median RS for patients with EGFR mutation is 23.5 month, which is more than twice that (11.2 months) for patients without EGFR mutation, that is, EGFR wild type.

### Rectal Cancer

The therapy for rectal cancer has changed within the past 30 years as it concerns surgery, radiotherapy, and systemic therapy.

**Figure 11:**
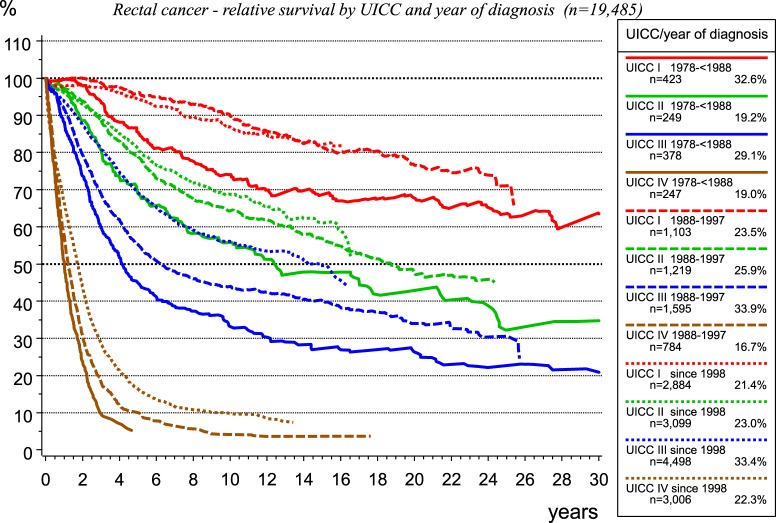
Relative survival for rectal carcinoma by UICC stage and year of diagnosis.

In the last decades, there has been a population-based implementation of total mesorectal excision of rectal cancer, along with a quality assessment using the MERCURY classification, as well as initially the implementation of adjuvant radiotherapy and subsequent of neoadjuvant radio-chemotherapy for UICC-stages II and III. Effectiveness of therapeutic innovations may be attested by data from population-based CCRs, as presented in Figure 11, for rectal carcinoma treated within the catchment area of the MCR.

## Knowledge Acquisition

### Incidence of Second Malignancies

**Figure 12:**
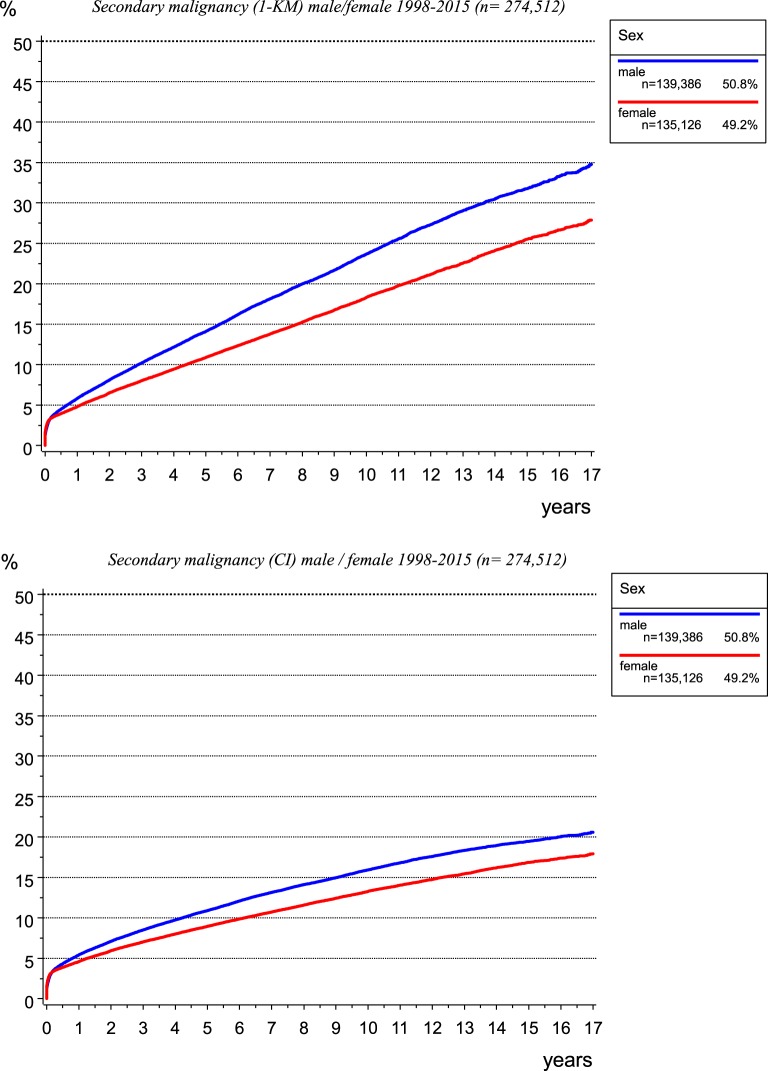
Risk of secondary malignancy as calculated using the Kaplan–Meier method (left) and cumulative incidence function when accounting for competing risks (right).

The incidence of second malignancies is not well known. The network of different medical departments for different tumor entities within a cancer registry enables gathering all malignancies. Thus, multiple malignomas can be compiled, which is difficult for a single department of a particular discipline. In addition, results of risk estimations depend on the calculation method. Whereas the probability calculated using the Kaplan–Meier method considers the cases lost to follow-up due to censoring, the inverse rate (1-KM) quantifies the percentage of secondary primaries occurring per year and cumulated over years (Figure 12, upper diagram).

The calculation of the cumulative incidence function (CI) considering competing risks (14), e.g., the risk of dying before a second malignancy is diagnosed, leads to lower probabilities of the occurrence of second malignancies (Figure 12, lower diagram).

### Translational Research

Translational medicine describes the effort, in which research results are transformed into routine patient care. One aspect in this field is the investigation of molecular characteristics and the functioning of cancer cells and its metabolites. The cooperation of the MCR with pathological institutes has led to a series of publications (15–23). Moreover, the connection between *in vitro* results and clinical data from CCRs validates laboratory insights regarding the course of the disease, the quality of life, and the prognoses.

### Hypothesis: Lymph Nodes do not Metastasize

The influence of positive lymph nodes on the process of metastasization is not finally clear and is one subject of research at the MCR. Although the presence of positive lymph nodes is a key prognostic factor, there is little evidence as to whether tumor cells in positive lymph nodes infiltrate other lymph nodes or distant organs. Moreover, while there is no evidence in the registry data of increased survival resulting from lymph node dissection. The success of the sentinel lymph node concept for some solid tumors and the fact that lymph node recurrence is rare in the course of disease of many solid tumors support the hypothesis, that “positive lymph nodes do not metastasize” (24).

## Limitations and Chances

Clinical cancer registries with their network for information processing and feedback provide a powerful infrastructure for optimizing patient care and initiating research projects. Though there are promising activities in the use of CCRs, the data are observational and thus contain various types of bias that must be considered in statistical analyses. Furthermore, the results should be interpreted with caution and knowledge of the state of the art, and the limitations and risks associated with using observational data must be evaluated separately according to the specific application.

Clinical cancer registries meet the demands of a defined catchment area, including population-based data attained due to the thoroughness of the registration and the appropriateness of the form and content of the incoming documents. Involvement of clinicians and scientists into cancer registries is necessary to keep registries in a current state and to support analysis of open questions. Therefore, catchment area for a population-based CCR should not cover far more than five million residents. Thus, it is highly likely that meaningful cancer data will be gathered and available for analyses by clinicians, scientists, and epidemiologists.

The main issue with respect to oncology and public health is the creation of transparency in patient care, developing state of the art updates in diagnostics and therapy, quantifying the outcome of procedures subject to the guidelines and, if necessary, defining starting points for improvement. The results from CCRs may be compared with those of other hospitals, with the results of randomized controlled trials, and with the results published in the national and international literatures. Positive and negative deviations must be noticeable and considered for drawing relevant conclusions. All this aspects until now are handled only partly or only in some regions but not for Germany in total. So, it will be a main task for CCRs to create an infrastructure and the valid database to deal with these questions on a regional, but comparable way.

While, in the past, many centers build up single databases without network and communication, such effects as described in the present manuscript offer significant short- and long-term benefit for all participants, generate large and multicenter data, and provide a comprehensive platform for scientific work as well as quality-related evaluations (25, 26).

The current work discusses only some of the applications and multiple aspects of the use of CCRs. The legislative and financial support provided by the KFRG should be used for further activities in health-care delivery research. Cancer control and patient care may benefit. In the future, such data will become even more important and will be an indispensable key element of all cancer centers.

## Author Contributions

GS-F: conception and design, collection and assembly of data, data analysis, and interpretation, manuscript writing, final approval of manuscript. SC and TK: conception and design, collection and assembly of data, final approval of manuscript. VN: collection and assembly of data, final approval of manuscript. JE: conception and design, collection and assembly of data, data analysis and interpretation, manuscript writing, final approval of manuscript.

## Conflict of Interest Statement

The authors declare that the research was conducted in the absence of any commercial or financial relationships that could be construed as a potential conflict of interest.
